# Testing Moral Disengagement and Proteus Effect Predictions on Feelings of Guilt and Self-Empowerment Attributed to Bearing Guns

**DOI:** 10.3389/fpsyg.2021.695086

**Published:** 2021-07-16

**Authors:** Ke M. Huang-Isherwood, Jorge Peña

**Affiliations:** Department of Communication, University of California, Davis, Davis, CA, United States

**Keywords:** moral disengagement, Proteus effect, guilt, gun perceptions, shooter video game, gang avatar, police avatar

## Abstract

This study (179 participants, mean age 19. 98, 85% female) examined how violence justification via avatar role manipulations affected first-person shooter game players' subsequent feelings of guilt and self-empowerment attributed to bearing guns in the real-world. In support of the moral disengagement in violent video games model, an independent samples *t*-test suggested that participants assigned to play as gang members shooting at police officers felt guiltier than those assigned to play as police officers shooting at gang members. In support of Proteus effect predictions linked with self-perception and priming mechanisms, a one-way repeated measures analysis of variance suggested that self-empowerment attributed to carrying guns for both avatar roles increased from baseline to after gameplay, but avatar roles did not influence the increase. The lack of influence could be because participants did not adopt avatar behaviors with undesirable connotations. The results highlight avatar-user bonds through which the associations raised by virtual personas affected players' emotions and self-perception when engaging in simulated violence.

## Introduction

Gun violence is commonplace in video games. For example, three out of five best-selling games in 2019 prominently feature gun violence: *Call of Duty: Black Ops 4, Red Dead Redemption 2*, and *Grand Theft Auto 5* (Entertainment Software Association, 2019). Technological advancements have made first-person shooter (FPS) video games, which directly pair players with gun violence, more realistic, and immersive (Lachlan and Maloney, 2008; McGloin et al., 2015). Current research is still divided as to the consequences of video game gun violence. While some researchers contend that video game gun violence contributes to real-life violence (Anderson and Bushman, 2002; Bushman et al., 2015), others dispute this link and find weak evidence for video game violence as a risk factor to real-life violence (Ferguson and Rueda, 2010; Ferguson, 2015).

Though the focus on whether video game violence contributes to real-life violence continues (Grizzard et al., 2016; Allen and Anderson, 2021), there is also interest in how violent video games influence moral disengagement or how players may display less aversive feelings after committing violent or immoral acts in a video game (Bandura, 1999; Hartmann and Vorderer, 2010). Moral disengagement is consequential because it leads players to become desensitized to violence (Grizzard et al., 2016) and to objectify human characters (Krcmar et al., 2014; Allen and Anderson, 2021). In particular, guilt—feelings about the consequences of a negative act, such as feeling that oneself has done something wrong—is a key emotional response that may be triggered and attenuated by violent video game play (Hartmann and Vorderer, 2010; Hartmann et al., 2010; Grizzard et al., 2016; Hartmann, 2017). This study aims to examine moral disengagement by investigating how playing a violent game in morally unjustified or justified character roles influence feelings of guilt.

More specifically, this study examined how playing as a game character with or without moral justification to use violence (i.e., police officer vs. gang member) influences feelings of guilt among video game players. Police officers and gang members are archetypal heroes and villains of many forms of media, and are often portrayed as “good guys” and “bad guys” (Eden et al., 2015; Bushman, 2018). In the U.S., these archetypes originated from the Western genre, in which police officers originated from Caucasian cowboys who act as law enforcers and gang members originating from people of color (especially Native Americans) who, disparagingly, represented the uncivilized (Ames, 1992). In addition, the image of police officers somewhat overlaps with military forces. Both police officers and military forces are uniformed and bear firearms (Bushman, 2018). SWAT forces particularly blur the line between the police and the military, due to the SWAT using equipment and weapons similar to those of soldiers (Kraska, 2006). Some modern examples of police and gang representation in the media include the *Lethal Weapon* TV series, the *Grand Theft Auto* (*GTA*) game series, and the international streaming service series *Narcos* (Ames, 1992; DeVane and Squire, 2008; Egner, 2015; Miller, 2016). The development of the *GTA* series reveals an interesting self-contained and influential role of media archetypes about police officers and gang members. Although *GTA* ostensibly takes place in a fictionalized Los Angeles, California, the developers from Dundee, Scotland, were mainly inspired by earlier media portrayals, rather than real-life personal experiences or research about crime and law enforcement in L.A. (DeVane and Squire, 2008).

The present study also attempts to increase the depth of the literature on psychological avatar-user bonds by examining how playing a violent game as a gang member vs. police officer avatar may influence players' self-empowerment attributed to carrying guns or beliefs about how carrying a gun makes players feel stronger, safer, and more powerful (Shapiro et al., 1997). On this regard, the Proteus effect predicts that avatar appearance and role may then steer players' self-perception (Yee and Bailenson, 2007), exert priming effects (Peña et al., 2009), or both (Ratan et al., 2019), which increase the salience of known stereotypes and concepts. However, individuals may be less likely to assimilate to avatars with negative social connotations (McCain et al., 2018). Considering the above, this study examines the yet untested prediction that playing a violent game from the perspective of a gang member or police officer will influence players' subsequent beliefs regarding how carrying a gun makes them feel strong and powerful.

## Moral Disengagement

In order to understand the how avatar-user bonds can influence players' feelings of guilt, we turn to moral disengagement processes associated with mediated experiences. This mechanism attempts to explain how mediated behaviors that usually would violate morality in real life may become permissible or even desirable when playing video games. Hartmann (2011, 2017) postulated that the processes are dual in nature, with an experiential (or automatic) and a rational (or reflective) component. The experiential component is faster and utilizes less cognitive energy, while the rational component is slower and expends more energy (Hartmann, 2011). Bandura (1999) explicated eight specific ways moral disengagement can occur, which later scholars summarized it as being related to reframing morally suspect actions as more positive, reducing individual responsibility over and negative consequences of the immoral actions, and reframing nature and role of the victims to make them more blameworthy (Allen and Anderson, 2021). Moral disengagement may occur when one or more of the processes above occurs (Allen and Anderson, 2021). One form of moral disengagement involving some of these processes is justification of violence, which refers to the extent to which individuals find it necessary and valid to physically harm another person (Hartmann et al., 2010). Violent virtual behavior then is not necessarily limited to “bad” game characters, even “good” game characters may behave violently (Eden et al., 2015; Hartmann, 2017). Individuals may understand violence as being justified if it is for a greater good, such as when game narratives involve saving the world or restoring justice (Hartmann et al., 2010).

Moral disengagement processes associated with mediated experiences assume that interactions with media are similar to social interactions in real life (Reeves and Nass, 1996; Hartmann, 2017). In particular, moral disengagement decreases perceived guilt, which refers to an aversive emotion stemming from violating societal or self-imposed morals (Hartmann, 2011, 2017). Guilt may result from reactions to the consequences of violating morals (e.g., causing harm; breaking norms, traditions, or laws; fear of punishment, etc., Caprara et al., 1992; Grizzard et al., 2016). Note that guilt relies on higher levels of rational or reflective involvement, because it is more intricate. Unlike basic emotions such as fear and anger, guilt relies on reflective recognition that one has transgressed a prohibition and on cognitive awareness of the consequences of the transgression (Grizzard et al., 2016).

In video games, guilt can be increased by manipulating game opponents, game avatars, avatar actions, and game environments. For example, older game visual and auditory characteristics, such as those less naturalistic classic arcade games like *Space Invaders*, may be less alike real life and utilize more player cognitive effort (Hartmann, 2011). As another example, just providing more personal background information of game opponents results in higher player guilt outcomes, likely due to players perceiving their opponents as more individualized and unique (Hartmann et al., 2010, study 2). Even game environments involving participant avatars acting violently toward non-player characters can increase guilt if the participants consider the environment more similar to real life (Weaver and Lewis, 2012). Other factors that may decrease guilt include distancing oneself from a game avatar's actions (Allen and Anderson, 2021), portraying game opponents as blameworthy (Hartmann and Vorderer, 2010), and when enemy characters are perceived as non-human (Krcmar et al., 2014).

Additionally, experimentally manipulating violence justification using game roles may affect players' feelings of guilt (Hartmann and Vorderer, 2010; Hartmann et al., 2010). In an experiment using a modified version of the FPS *Operation Flashpoint*, players randomly assigned to the role of a United Nations soldier who used violence to save innocents from torture and imprisonment reported less guilt than those assigned to a paramilitary soldier role who used violence to defend imprisonment and the torture camp (Hartmann and Vorderer, 2010). In a follow-up study, participants were randomly assigned to repeatedly play the modified *Operation Flashpoint* for 4 days after assignment in justified or unjustified violence roles (Grizzard et al., 2016). On the fifth and last day of the study, researchers assigned all participants to play as terrorists in *Call of Duty*. Participants assigned to unjustified violent roles reported increased guilt relative to their justified violence role counterparts in the first 4 days and, additionally, repeated play in an unjustified violent game role led players to feel less guilty when playing as terrorists in the new game (Grizzard et al., 2016). In addition, playing as a character that performed moral actions (i.e., helping and protecting game characters in *Fallout 3*) compared with playing as a character that committed immoral actions (i.e., hurting and killing game characters) resulted in increased feelings of guilt and shame, especially among players who were more transported by the game narrative (Mahood and Hanus, 2017). Finally, playing as a protagonist who exterminates evil creatures (i.e., poisonous, insect-like creatures in a modified version of *The Elder Scrolls V: Skyrim*) compared with a protagonist who exterminates innocents (i.e., townspeople, children, and dogs) resulted in higher feelings of guilt (Allen and Anderson, 2021).

Given this literature as background, this study contributes to the moral disengagement literature by examining how the archetypical image of gang member avatar may lead to higher perceived guilt due to lower moral disengagement relative to using police officer avatars. Specifically, the archetypal image of police officer avatars may lead to lower perceived guilt due to increased perceived justification to use violence relative to the image of gang member avatars. Specifically, players assigned to police avatars may have automatic images that their virtual violent action is justified by a greater good and that the gang members are to blame.

*H1:* Participants who play a violent game in the role of a gang member character will feel guiltier than those who play a violent game as a police officer.

## The Proteus Effect

The Proteus effect refers to a specific avatar-user bond in which the appearance and role of a user's digital body directly influences their cognition and behavior. It predicts that individuals may conform to the stereotypical connotations inferred from their avatar. The Proteus effect has been linked to self-perception, priming mechanism, and both. From a self-perception perspective, individuals may draw inferences about the self-based on spontaneous past behavior (Yee and Bailenson, 2007). In support of these assumptions, participants randomly assigned to operate physically attractive avatars stood closer and disclosed more information in a conversation relative to those assigned to unattractive avatars (Yee and Bailenson, 2007). From a priming perspective, avatar appearance and roles can activate known concepts and stereotypes stored in memory and as such individuals displayed stereotype-consistent thoughts and behavioral scripts (Peña et al., 2009). For instance, participants assigned to thin instead of obese avatars showed more physical activity while playing a motion-controlled tennis game (Peña et al., 2016). Relative to participants assigned to young avatar or a control group, participants who had earlier embodied older avatars took longer to walk a set distance (Reinhard et al., 2020).

Though the Proteus effect has been tested in several contexts and displays a reliable small to medium statistical effect size (Ratan et al., 2019), studies have not yet examined whether the associations raised by avatars can influence individual perceptions about guns in the real world. In particular, self-empowerment attributed to bearing guns refers to the perception that firearms strengthen armed individuals (Shapiro et al., 1997; Matson et al., 2019). Self-empowerment attributed to bearing guns may stem from the belief that firearms are useful for controlling threats (Shapiro et al., 1997; Matson et al., 2019). Of key importance to this study, media consumption can influence attitudes toward guns. For example, heavy viewership of crime television dramas was associated with the belief that carrying guns can best prepare individuals for self-defense (Dowler, 2002). Moreover, greater experience with video game gun controller use was associated with increased support for the instrumental utility of guns, though FPS playing frequency was not correlated with the greater support for guns' instrumental utility (Lapierre and Farrar, 2016). Participants who played *Time Crisis 4* with gun replica game controllers showed increased perceptions of realism, controller naturalness, immersion, and cognitive aggression compared with those who played with traditional button and joystick controllers (McGloin et al., 2015). In addition, aggressive thoughts were more accessible for participants who were exposed to photos of people shooting at other people relative to those exposed to photos of people shooting at inanimate objects and photos of people without guns (Bushman, 2018, experiment 2). Moreover, participants assigned to play with Black avatars that targeted White enemies in a third-person shooter game selected more difficult geometrical figures to a White individual in the real-world relative to participants assigned to play with Black avatars targeting Black enemies (Hawkins et al., 2021). This implies increased post-game aggressive behavior in intergroup instead of ingroup conditions. Relative to those who played as a Black avatar against Black enemies, participants who played with White avatars also showed increased motivation to harm a White partner when game enemies were Black (Hawkins et al., 2021).

Considering that mediated experiences may affect perceptions about guns, it is possible that playing a shooter game as police and gang member avatars may increase self-empowerment attributed to bearing guns relative to baseline scores. Such prediction is congruent with the self-perception mechanism in the Proteus effect (Yee and Bailenson, 2007), which anticipates that unprompted overt behavior, such as shooting guns as police officers or gang members in a game may influence individuals' attitudes. This prediction also agrees with the priming mechanism in the Proteus effect (Peña et al., 2009), which expects that the associations about gun empowerment raised in a shooter game when playing in the role of police officers or gang members may transfer to subsequent situations. Thus:

*H2:* Relative to baseline scores, participants will show increased self-empowerment attributed to bearing guns after playing a violent game in the role of police officers and gang members.

Though moral disengagement mechanisms have not been directly linked to the Proteus effect, there is evidence that individuals are less likely to adopt the behaviors of avatars with undesirable connotations. For example, participants did not take on the luxury purchase behaviors after embodying an avatar representing a narcissistic celebrity relative to those who embodied a generic avatar (McCain et al., 2018). This hypothesis needs to be further tested as there is conflicting evidence implying that individuals may also exhibit the behaviors of avatars with negative connotations. For instance, participants show increased violent intentions after controlling avatars in dark instead of light uniforms (Peña et al., 2009, experiment 1). In addition, participants assigned to Ku Klux Klan-like avatars generated more aggressive spontaneous stories in reaction to ambiguous drawings relative to those assigned to doctor and transparent avatars (Peña et al., 2009, experiment 2). Moreover, participants show increased aggressive behavior after playing a game using villainous instead of heroic and control avatars (Yoon and Vargas, 2014). Based on moral disengagement and Proteus effect assumptions, we predict that:

*H3:* Relative to baseline scores, participants assigned to play a violent video game in the role of police officers will show increased self-empowerment attributed to bearing guns relative to those assigned to play as gang members.

## Method

### Participants

One hundred and eighty-nine undergraduate students at a large U.S. West Coast university participated in the study for extra credit. The data were collected in 2019. Thus, the findings were not influenced by the 2020 movement for police restructuring and defunding. Ten participants were disqualified from the study because they showed high suspicion of the study's purpose, reported feeling unwell, or experienced technical difficulties. Out of the remaining 179 participants (gang members = 90; police officers = 89), 85% were female, their ages ranged from 18 to 54 (*M* = 19.98, *SD* = 3.51), were from all class standing levels (44% Freshmen, 21% Sophomore, 25% Junior, and 10% Senior), and liberal leaning (1 = *extremely liberal*, 7 = *extremely conservative*; median = slightly liberal). Table 1 lists more detailed descriptive statistics and statistical comparisons of the demographic information, offering support for the study's internal consistency.

**Table 1:** Descriptive statistics and statistical comparisons of the avatar groups.

	**Both avatars (*n* = 179)**	**Gang member avatar (*n* = 90)**	**Police officer avatar (*n* = 89)**	**Comparisons of the gang members vs. police officers**
Gender	Males = 26 Females = 152 Non-binary = 1	Males = 13 Females = 77 Non-binary = 0	Males = 13 Females = 75 Non-binary = 1	$\chi_{\left(2\right)}^{2}$ = 1.02, *p* = 0.600
Age	*M* = 19.98, *SD* = 3.51	*M* = 19.63, *SD* = 1.45	*M* = 20.24, *SD* = 4.74	*t*_(104.13)_ = −1.34, *p* = 0.183
Class standing	Freshmen = 78 Sophomore = 38 Junior = 45 Senior = 18	Freshmen = 39 Sophomore = 21 Junior = 24 Senior = 6	Freshmen = 39 Sophomore = 17 Junior = 21 Senior = 12	$\chi_{\left(3\right)}^{2}$ = 2.62, *p* = 0.455
Political ideology	*M* = 2.93, *SD* = 1.26	*M* = 2.94, *SD* = 1.35	*M* = 2.92, *SD* = 1.17	*t*_(173.94)_ = 0.12, *p* = 0.903

*Political ideology (1 = extremely liberal, 7 = extremely conservative). M, mean; SD, standard deviation; n, sample size. Statistical comparisons in independent samples t-tests for continuous variables and chi-square tests for categorical variables*.

Assuming a small to medium effect size of Cohen's *d* = 0.30 on the relationship between violence justification and guilt similar to Hartmann and Vorderer (2010) and Ratan et al. (2019), we conducted a sensitivity power analysis (with α = 0.05) on G^*^Power 3.1.9.4 (Faul et al., 2007). The power analysis indicated that the participant sample size was sufficient to detect the small to medium effect with 0.80 power in a *t*-test, as at least 75 participants were necessary per condition to detect a significant effect.

### Stimulus

**Figure 1:**
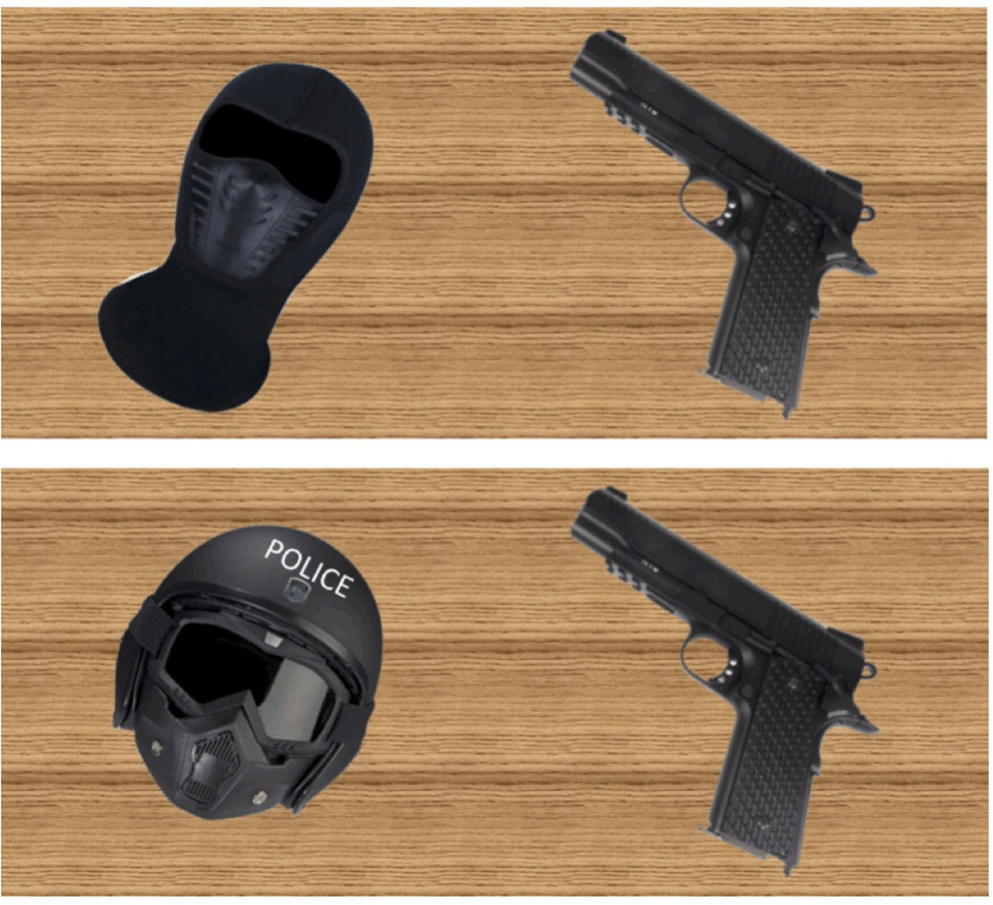
A comparison of a zoomed-in cutscene screenshot showing what participants saw of themselves, that is, assigned gang members and their equipment (**Top** image) and assigned police officers and their equipment (**Bottom** image).

A video game was custom-made to test for the hypotheses above. The game was conceptualized and overseen by the authors, and it was programmed by four undergraduate students using the Unity platform and its assets. The video game featured a short cutscene with the game's story. It introduced a large luxury department store in a dangerous city, with the gang member game version presenting a story that the player was a criminal caught stealing and then fighting off armed police officers (unjustified violence). In the police officer game version, the player was on duty investigating a theft and then fighting against armed gang members (justified violence). The introductory cutscene and the game itself were identical across conditions except for the experimental manipulations. The face, body, and skin color of the game character operated by participants was not shown. Figure 1 shows the main avatar manipulation in the cutscene. In both versions, gang member game enemies were Caucasian. This was done to avoid biases linked to an increased likelihood of shooting dark-skinned enemy characters (Correll et al., 2007). In both versions, the enemies wore black tactical clothes. In the gang member avatar condition, enemies had the word “POLICE” affixed on their clothes. Enemies did not have “POLICE” affixed to their clothes in the police member avatar condition.

**Figure 2:**
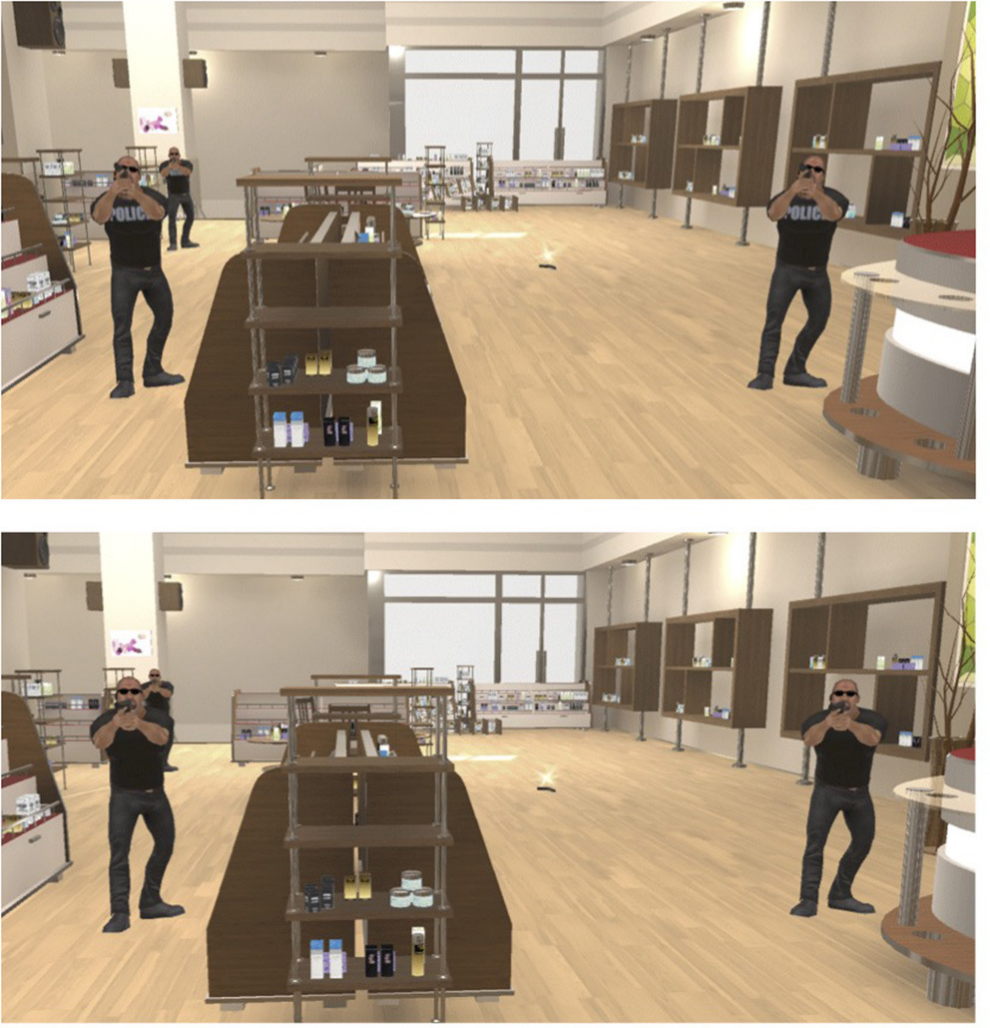
A comparison of a zoomed-in game screenshot showing what participants saw of their enemies, that is, assigned gang members (whose enemies are police officers, **Top** image) and assigned police officers (whose enemies are gang members, **Bottom** image).

The game tasked players to shoot and kill their enemies while avoiding getting shot. Players had to kill their enemies in order to collect ammunition and advance in the game. The game discouraged players to be reckless with their ammunition and health. If the players' health deteriorated, the screen showed larger blood spots that impeded vision and the game played faster heartbeat sounds. In order to increase tension, onscreen blood spots made it more difficult for players to see and aim. However, the game did not allow players to die, and the onscreen blood spots slowly disappeared if they avoided being hit by enemy fire. This allowed participants to experience high-paced action and a degree of risk when playing the game, while also allowing for participants with less shooter game experience—who often partake in lab studies (Hartmann and Vorderer, 2010; Krcmar et al., 2014)—to participate in the study with less frustration. The game ended when the player killed all of the enemies and passed through the department store exit. Figure 2 shows the two versions of the game environment. Both versions of the game are available at https://osf.io/p8cnq/?view_only=246dc4e4fa5848e1acfefef12292593a.

### Procedure

Ethical approval was granted by the Institutional Review Board Administration office of the participants' university. The participants were recruited through the university's online social science research administration system. Participants consented to partaking in this study and then completed an online questionnaire with demographics measures and a self-empowerment attributed to bearing guns baseline items. The questionnaire also contained gun policy and demographic measures that were reported in a separate study (Huang-Isherwood, 2020). After the questionnaire but still in the same school term (*M* = 19.82 days, *SD* = 15.20 days), participants scheduled an in-person lab appointment. To avoid self-selection based on pre-existing video game preference, the study's online recruitment and scheduling page in the administration system did not specify the topic or procedure of the study; it only mentioned its time and location. The baseline questionnaire and the experiment took place in different days because the questionnaire contained sensitive questions about bearing guns and gun policy that may have increased participant suspicion and raised their awareness about the study's hypotheses. This procedure has been applied in other studies on sensitive topics (e.g., Bailenson et al., 2008).

At the in-person lab appointment, a lab research assistant welcomed each individual participant and, to mask the purpose of the study, the assistant told the participant that the session was to test materials for future research. Lab assistants followed a previously prepared random assignment master list spreadsheet, which included an ordered number list (1, 2, 3,… 200) and the corresponding random avatar assignment (police, gang, gang,… police). Participants were assigned to the avatar according to their order of arrival to the lab appointment. Prior to the participants' arrival, lab assistants opened the corresponding version of the game. The assistants timed participants as they played. Consistent with other studies, participants played for up to 15 min (e.g., Grizzard et al., 2016; Mahood and Hanus, 2017). If a participant finished before 15 min, assistants recorded the time and allowed participants to proceed. This was to ensure that more skilled participants were not bored or frustrated for having to replay the game. Still, participants played for almost 15 min (*M* = 14:17 min, *SD* = 1:44 min). After playing the game, participants answered a questionnaire with their recollection of their assigned role, feelings of guilt, again the self-empowerment attributed to bearing guns, and shooter game experience. Assistants debriefed the participants and awarded them with an extra credit. The complete study took about 90 min.

### Measures

#### Feelings of Guilt

We employed a three-item guilt as a state measure that was used in studies examining guilty feelings after playing video games (e.g., Hartmann and Vorderer, 2010). The measure asked if the participant, while playing the game, felt “regret,” “sorry about something you've done,” and “like you've done something wrong” (Hartmann et al., 2010) on a 5-point Likert-type scale (1 = *Rarely or never*, 5 = *Very often*). The items were reliable (Cronbach's α = 0.92) and hence were averaged into a single score.

#### Self-Empowerment Attributed to Bearing Guns

We adapted items from the attitudes toward guns and violence questionnaire (Shapiro et al., 1997) to measure participants' self-empowerment attributed to carrying guns before and after the playing the experimental game. The items measured participant's level of agreement/disagreement that “carrying a gun makes me feel strong,” “bearing a firearm can increase my sense of power,” “carrying a gun makes me feel safe,” and “bearing a firearm can increase my sense of safety” on a 17-point Likert-type scale (1 = *Strongly disagree*, 17 = *Strongly agree*). The decision to employ a 17-point scale recognizes the need to be sensitive to the participant sample attitudes (Blanton and Jaccard, 2006). The participant sample involved students from a left-leaning West Coast university and most of the sample had anti-gun attitudes, more concretely, negative attitudes about the action of carrying/bearing guns (Miller et al., 2002). In addition, more than half of the participants self-identified as being liberal. In this context, using a 17-point bipolar rating, instead of the more common 5- or 7-point bipolar rating allowed for more variability at the ends of the scale (i.e., 1–8 range or 10–17 range). For example, Burrows and Blanton (2016) conducted three studies to examine the effect of anti-drunk driving ads embedded in a game on participants' willingness to drive under the influence (DUI) of alcohol, a socially undesirable behavior. This study employed 17-point scales to be able to capture subtle variability in DUI attitudes. Indeed, for both the baseline and the post-gameplay measurements of self-empowerment attributed to bearing guns, the averages leaned toward “disagree” end of the scale (see Table 2). The pre and post-scales were reliable (Cronbach's α_Time1_ = 0.90; Cronbach's α_Time2_ = 0.93) and were thus averaged into corresponding baseline and post-gameplay self-empowerment attributed to bearing gun scores.

**Table 2:** Descriptive statistics of the main variables of interest.

	**Both avatars**		**Gang member**		**Police officer**	
	***M***	***SD***	***M***	***SD***	***M***	***SD***
Feelings of guilt	2.26	1.19	1.90	1.07	2.51	1.22
**Self-empowerment attributed to bearing guns**						
Time 1	5.45	3.98	5.05	4.03	5.66	3.94
Time 2	6.79	4.43	6.16	4.43	7.43	4.35
Shooter game experience	1.65	1.04	1.65	1.02	1.66	1.06

*M, mean; SD, standard deviation*.

#### Shooter Game Experience

We employed a two-item shooter game experience measure to capture the participants' background on playing such games (Matthews, 2011), due to the possible cumulative effects of repeated play of shooter games (Grizzard et al., 2016). The measure offered contemporary popular examples of shooter games (*Call of Duty, Battlefield, Halo 5: Guardians*) and asked the participant their play “frequency” and “on higher difficulty than default” (Matthews, 2011) on a 5 Likert-type scale (1 = *Never*, 5 = *Frequently*). The items were reliable (Pearson's *r* = 0.75, *p* < 0.001) and hence were averaged into a single score.

### Statistical Analysis

We first planned to conduct a manipulation check by testing actual avatar role assignment with participant recollection of their assigned role. To examine H1, we planned to carry out an independent samples *t*-test of the participant assigned role on feelings of guilt. To examine the remaining hypotheses, we planned to carry out a one-way repeated measures analysis of variance (ANOVA) to test for the differences between the baseline and post-gameplay self-empowerment attributed to bearing guns (H2) and the interaction between increase over time and role assignment (H3). All analyses were conducted in SPSS 27.

## Results

Table 2 presents the descriptive statistics of the main variables of interest, specifically means and standard deviations.

To ensure that the violence justification manipulation was successful, we evaluated whether participants correctly recalled their assigned gang member or police officer avatar role. A comparison of actual role assignment against participant self-reported assignment found that most participants correctly recalled that they were assigned to play as a gang member (*n* = 87, 97%) or as a police officer (*n* = 78, 88%). A chi-square test revealed that comparing the actual avatar role assignment against recollection of the role assignment did not vary by experimental condition, $\chi_{\left(1\right)}^{2}$ = 0.21, *p* = 0.650, thus suggesting no differences in avatar role assignment recall between the conditions. Because a small proportion of participants did not correctly recall their role assignment, the data analyses included all participants who completed the study, as participants could have unconsciously perceived their avatar's role even if they did not explicitly report it (O'Keefe, 2003; Hartmann and Vorderer, 2010). Furthermore, (https://osf.io/p8cnq/?view_only=246dc4e4fa5848e1acfefef12292593a) report data on whether correct or incorrect recollection of assignment condition was a relevant covariate and found no significant differences.

To test for whether players randomly assigned to play as gang members would feel guiltier than the players assigned to play as police officers (H1), we carried out an independent samples *t*-test of the participant assigned condition on feelings of guilt. Indeed, participants assigned to the gang member avatar role (*M* = 2.51, *SD* = 1.22) show increased feelings of guilt relative to those assigned to the police officer version (*M* = 1.90, *SD* = 1.07), *t*_(174.46)_ = 3.55, *p* < 0.001, Cohen's *d* = 0.53. Thus, H1 was supported. Furthermore, to check if shooter game experience altered the finding, we conducted an analysis of covariance (ANCOVA) of assigned role on feelings of guilt with shooter game experience as the covariate. We found similar results with only assigned role as being statistically significant on guilt, *F*_(1,176)_ = 12.73, *p* < 0.001.

To test for baseline and post-gameplay self-empowerment attributed to bearing guns (H2) and the interaction between increase over time and avatar assignment (H3), we carried out a one-way repeated measures ANOVA. As expected, there were no statistically significant differences in baseline scores between participants assigned to gang member and police officer avatars, *t*_(173.92)_ = −0.68, *p* = 0.500. The results of the one-way repeated measures ANOVA show that self-empowerment attributed to bearing guns significantly increased from baseline scores (*M*_Time1_ = 5.45, *SD*_Time1_ = 3.98) to post-game play (*M*_Time2_ = 6.79, *SD*_Time2_ = 4.43), *F*_(1,167.06)_ = 25.72, *p* < 0.001, Cohen's *f* = 0.38. Since Mauchley's test of sphericity was violated, the Greenhouse-Geisser correction was employed. Thus, H2 was supported.

However, there was no significant interaction between time (baseline, post-gameplay) and avatar assignment, *F*_(1,13.72)_ = 2.11. This analysis also employed the Greenhouse-Geisser correction. Thus, H3 was not supported. Furthermore, to check if shooter game experience altered the findings, we conducted a repeated measures ANCOVA with the same variables as the ANOVA above and shooter game experience as a covariate. We found similar results, with only a statistically significant increase from baseline scores to post-game play, *F*_(1,63.26)_ = 9.70, *p* = 0.002, and no other factors.

## Discussion

This study investigated how violence justification through video game avatar manipulations influenced players' feelings of guilt and self-empowerment attributed to bearing guns. The findings from hypothesis 1 supported the predicted moral disengagement processes in video games. Perceived guilt was higher for participants randomly assigned to play a custom-made shooter game as gang members robbing a store and shooting at police officers relative to those assigned to play as police officers shooting at gang members robbing the store. By focusing on these archetypal avatar roles in this scenario, this study extended previous studies that focused on the how player's game role and actions, such as committing virtual physical harm and homicide influenced perceived guilt (Hartmann and Vorderer, 2010; Hartmann et al., 2010; Weaver and Lewis, 2012; Grizzard et al., 2016; Mahood and Hanus, 2017; Allen and Anderson, 2021), to also apply to virtual property crimes (i.e., robbing luxury goods). In particular, it extended previous studies which focused on military and combat environments with avatars more frequently being soldiers in order to examine moral disengagement (Hartmann and Vorderer, 2010; Grizzard et al., 2016; Hartmann, 2017) to more civilian contexts. This initial contribution may serve as a starting point for future studies that employ police and gang avatar roles to examine desensitization—i.e., habituation to violence through repeated exposure—and real-life violent behavior outcomes. For example, longitudinal analyses may show whether individuals display increased desensitization in earlier game stages when embodying morally justified instead of justified roles. It is also possible that, at later stages, players assigned to both morally justified and unjustified game avatars experience low guilt. Based on Grizzard et al. (2016) findings, future studies should also test whether switching after repeated play from police officer avatar roles to a morally unjustified role, such as playing as a gang member, leads to decreased guilt relative to switching from gang members to police officers.

In addition, future studies should investigate the internal mechanisms in moral disengagement processes. In this study, it is unclear whether the justification for violence occurred due to moral reframing of the avatar self or of the actions of the game opponents. This could be tested by breaking apart the two conditions of this study into four: police officers shooting at opponents who are also officers, police officers shooting at opponents who are gang members, gang members shooting at opponents who are also gang members, and gang members shooting at opponents who are police officers. Examining guilt across these four conditions would allow a deeper understanding of whether the deciding factor is the nature of the avatar self or of the opponent.

Considering that cognitive awareness of the consequences of a moral transgression is needed to experience guilt (Grizzard et al., 2016), manipulating the amount of cognitive resources available to participants may be employed to test whether lack of cognitive resources influences how game-related moral justification affects perceived guilt. Manipulating the availability of cognitive resources could be done by asking participants to remember short or long digits (Peña et al., 2018; Read et al., 2018). One possibility is that the lack of cognitive resources could make players in unjustified avatar roles to be less likely to feel guilty because they may be temporarily unable to reflect on their actions. Alternatively, moral disengagement processes can occur with little cognitive effort, thus implying that mental overload may not influence player guilt (e.g., Krcmar et al., 2014; Eden et al., 2015).

This initial contribution has practical implications for both violent and non-violent serious games. Particularly, serious games with robbery scenarios that can help law enforcement and rehabilitation officials—police officers, judges, prosecutors, prison guards, wardens, probation officers, and parole officers—take on the perspectives of criminals and perceive guilt. These effects could help these officials better understand the point of view of criminals, avoid dehumanizing them, and reintegrate them to the wider society. Moreover, the benefit of such serious games is that the scenarios would be virtual and there would not be the same degree of risk and harm involved.

In regard to hypothesis 2, the study uncovered that relative to baseline scores, self-empowerment attributed to bearing guns was increased after playing an FPS game regardless of avatar role. From a Proteus effect standpoint, this was rooted in how both avatar conditions asked participants to use guns along with how avatar appearance primed aggressive constructs stored in the memory. This finding is also congruent with the General Aggression Model (GAM), which proposes that exposure to video game violence increases the accessibility of aggression-related knowledge structures (Anderson and Bushman, 2002). According to GAM, the increased accessibility of aggressive thoughts and emotions may lead to an augmented likelihood of resorting to violence as a viable response. This finding is also congruent with the Game Transfer Phenomena (GTP), which proposes that players, with different levels of susceptibility, can unconsciously replicate their in-game virtual world experiences to the real world, particularly through altered perceptions, sensations, cognitive processes, and behaviors (Ortiz de Gortari and Griffiths, 2016; Ortiz de Gortari, 2019). Of particular interest here are cognitive distortions from in-game shooting cues and repeated shooting tasks that carry over to perceptions about guns in the real world (Ortiz de Gortari and Griffiths, 2014, 2016).

The findings from hypothesis 3 did not suggest that controlling morally unjustified or justified avatars engaged in gun violence had different effects on self-empowerment attributed to bearing guns. Future studies could test whether controlling for other archetypes, such as soldier and vigilante avatars may specifically influence self-empowerment attributed to bearing guns. In order to further explore this effect and to separate Proteus effect from GAM predictions, future studies should assign participants to either control or watching police and gang member avatars enacting gun violence. Controlling avatars may have stronger effects than simply watching (Yee and Bailenson, 2009), and thus self-empowerment attributed to bearing guns may be more strongly influenced by controlling instead of watching avatars carrying on gun violence. This could also be achieved by implementing control conditions with no visible avatars in order to detangle the effect of playing as an avatar archetype from simply playing a given shooter game.

## Limitations

Despite this study's insights, there were limitations. Our sample was mostly college freshmen women, who are often not the target population of FPS games (Entertainment Software Association, 2020). Our measurement of shooter game experience further confirmed that the majority of the sample participants had little shooter gameplay experience. Additionally, our sample was mostly of students who had negative attitudes about carrying guns. Future studies could replicate this study, including samples with both anti- and pro-gun attitudes, to test whether the perceived guilt and gun empowerment findings remain the same. Future studies should also more directly address racialized violence by displaying police officer and gang member game characters as being racial majorities or minorities. In a study examining shooting decisions in a simulated environment, community members were more likely than police officers to mistakenly shoot at Black than Caucasian targets (Correll et al., 2007). In another study manipulating participants' avatar as being Caucasian or Black, relative to those with Caucasian avatars, those with Black avatars decreased their implicit bias against Black people, an effect that was maintained for at least 1 week (Banakou et al., 2016). Thus, future research could investigate whether the manipulation of enemy characters' skin color affects guilt and attitudes about gun self-empowerment.

## Conclusion

In conclusion, the moral justification of game avatars to use violence can influence players' feelings of guilt. This indicates how avatar-user relationships reliably influence emotions that require self-reflection and evaluation. In addition, the mere act of playing a shooter game increased self-empowerment attributed to bearing guns from baseline scores. Future research is needed to establish whether controlling different avatar archetypes that use guns can affect self-empowerment attributed to bearing guns.

## Data Availability Statement

Anonymized data supporting the conclusions of this article will be made available by the corresponding author, without undue reservation. Additional analyses and the game stimulus materials are available at https://osf.io/p8cnq/?view_only=246dc4e4fa5848e1acfefef12292593a an Open Science Framework page.

## Ethics Statement

The study was reviewed and approved by the UC Davis IRB Administration under IRBNet no. 1358880. The participants provided their written informed consent to participate in the study.

## Author Contributions

KH-I designed, collected data, analyzed data, and wrote up the study. JP advised and supported in all the aforementioned tasks. All authors contributed to the article and approved the submitted version.

## Conflict of Interest

The authors declare that the research was conducted in the absence of any commercial or financial relationships that could be construed as a potential conflict of interest.
